# The power of deep learning in simplifying feature selection for hepatocellular carcinoma: a review

**DOI:** 10.1186/s12911-024-02682-1

**Published:** 2024-10-04

**Authors:** Ghada Mostafa, Hamdi Mahmoud, Tarek Abd El-Hafeez, Mohamed E.ElAraby

**Affiliations:** 1 Computer Science Department, Faculty of Computers and Artificial Intelligence, Beni-Suef University, Beni-Suef, Egypt; 2 Computer Science Department, Faculty of Computers and Artificial Intelligence, Beni-Suef National University, Beni-Suef, Egypt; 3 Department of Computer Science, Faculty of Science, Minia University, EL-Minia, Egypt; 4 Computer Science Unit, Deraya University, EL-Minia, Egypt

**Keywords:** Deep Learning, Machine Learning, Hepatocellular carcinoma, Liver cancer, Feature selection, Artificial Intelligence

## Abstract

**Background:**

Hepatocellular Carcinoma (HCC) is a highly aggressive, prevalent, and deadly type of liver cancer. With the advent of deep learning techniques, significant advancements have been made in simplifying and optimizing the feature selection process.

**Objective:**

Our scoping review presents an overview of the various deep learning models and algorithms utilized to address feature selection for HCC. The paper highlights the strengths and limitations of each approach, along with their potential applications in clinical practice. Additionally, it discusses the benefits of using deep learning to identify relevant features and their impact on the accuracy and efficiency of diagnosis, prognosis, and treatment of HCC.

**Design:**

The review encompasses a comprehensive analysis of the research conducted in the past few years, focusing on the methodologies, datasets, and evaluation metrics adopted by different studies. The paper aims to identify the key trends and advancements in the field, shedding light on the promising areas for future research and development.

**Results:**

The findings of this review indicate that deep learning techniques have shown promising results in simplifying feature selection for HCC. By leveraging large-scale datasets and advanced neural network architectures, these methods have demonstrated improved accuracy and robustness in identifying predictive features.

**Conclusions:**

We analyze published studies to reveal the state-of-the-art HCC prediction and showcase how deep learning can boost accuracy and decrease false positives. But we also acknowledge the challenges that remain in translating this potential into clinical reality.

## Introduction

**Fig. 1:**
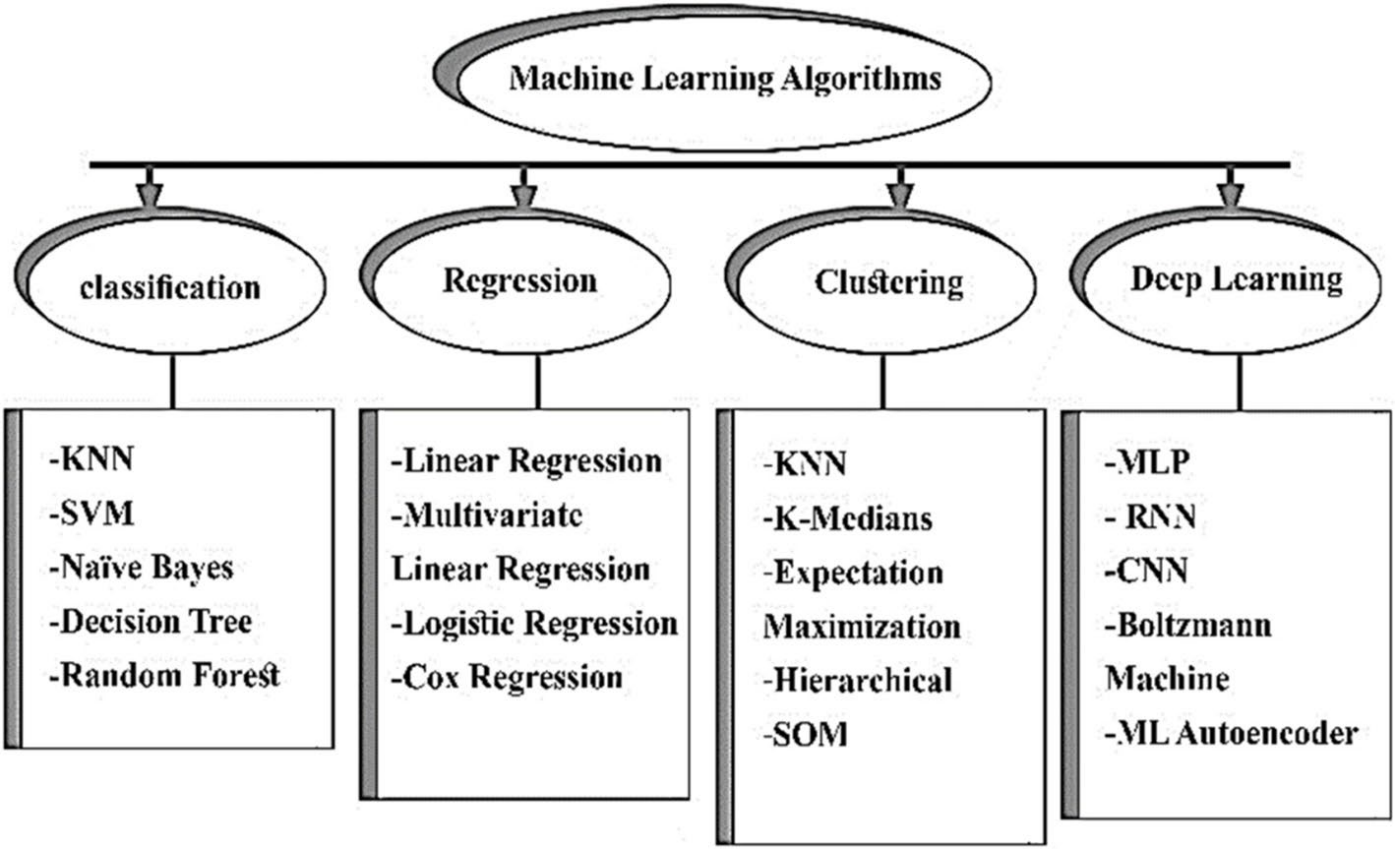
Illustration of machine learning and deep learning algorithms

Hepatocellular carcinoma (HCC) is a primary liver cancer that is characterized by its aggressive nature and often develops in individuals with chronic parenchymal liver diseases. It is currently ranked among the leading causes of cancer incidence and mortality worldwide [1, 2]. While effective antiviral therapy has led to a decline in the burden of HCC related to hepatitis B virus (HBV) and hepatitis C virus (HCV), the incidence of HCC associated with metabolic syndrome is expected to rise due to the significant increase in the prevalence of non-alcoholic fatty liver disease (NAFLD) in the general population [3]. Despite decades of research on HCC, the development of screening protocols, non-invasive diagnostic techniques based on imaging, and various treatment modalities such as surgical, locoregional, and systemic therapies, the overall prognosis for patients with HCC continues to be unfavorable. Patients with HCC face considerable unaddressed requirements in terms of predicting risks, detecting the disease at an early stage, accurately forecasting outcomes, and providing personalized treatment options [4, 5]. The volume of health data generated by patients with HCC is substantial, which presents a significant challenge in converting this data into useful knowledge for researchers. Artificial intelligence (AI) has emerged as a potential solution, as it possesses the capability to synthesize and analyze large amounts of multimodal data with high degrees of accuracy and reliability (Fig. 1). Given the knowledge vacuum in this area, we undertook a scoping review to lay the groundwork for future research by mapping existing studies and revealing critical gaps.

**Fig. 2:**
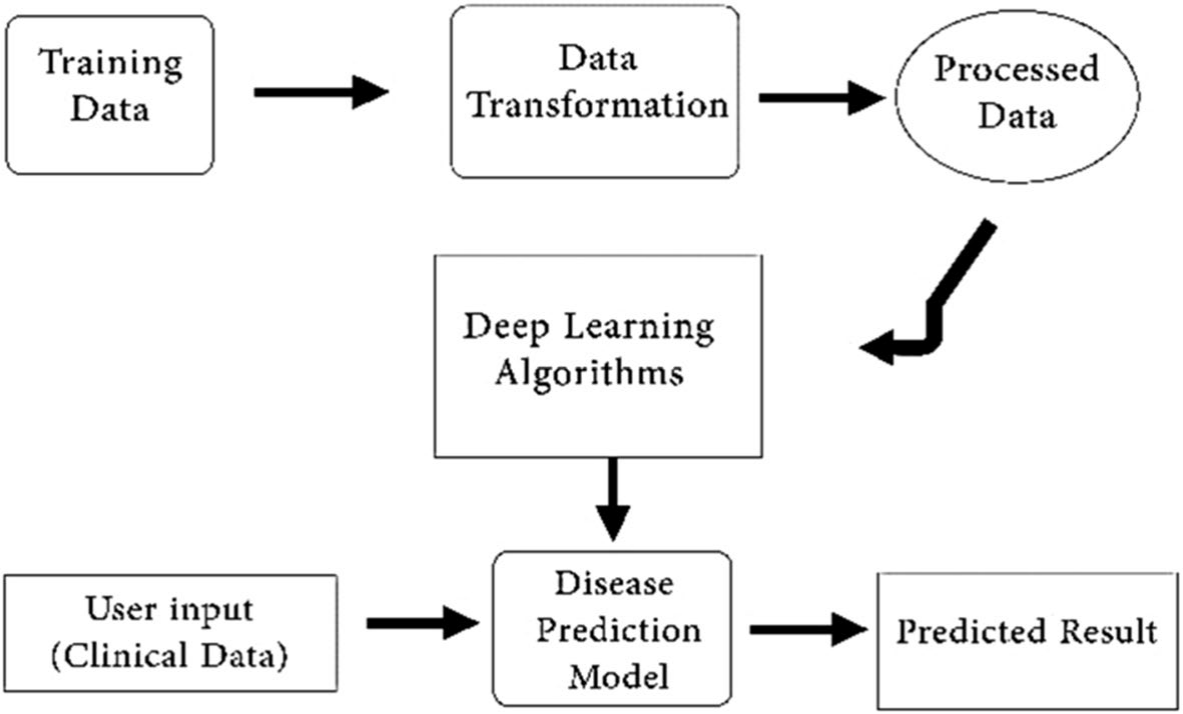
A simple framework for Disease prediction using deep learning algorithms

In recent years, AI has been increasingly applied to various medical fields, including hepatology, due to advancements in deep learning technology (Fig. 2). Deep learning algorithms can process a wide range of medical data, such as laboratory values, multi-omics studies, and medical imaging [6–8]. The objective of this review is to present a comprehensive overview of the potential uses of deep learning techniques in enhancing healthcare for patients with hepatocellular carcinoma (HCC) and provide examples of its application. Deep learning algorithms have shown promising results in risk prediction and diagnosis of cancer diseases, including the development of predictive models for HCC that can provide more accurate and reliable predictions than traditional methods [9, 10]. This review examines the existing research on predicting HCC using deep learning algorithms, including the types of algorithms used, datasets employed, and performance metrics reported, and also explores potential challenges and future directions for this field of research.

Deep learning techniques can be incorporated into the clinical practice for feature selection in the diagnosis and management of hepatocellular carcinoma (HCC) in the following ways:

1. **Automated Feature Extraction:** Deep learning models can be trained on large datasets of medical images (e.g., CT, MRI) and clinical data to automatically extract relevant features that are predictive of HCC. This can reduce the time and expertise required for manual feature engineering, which is often a labor-intensive and subjective process.
2. **Improved Diagnostic Accuracy:** Deep learning models have shown promising results in accurately identifying imaging biomarkers and clinical features associated with HCC. By incorporating these deep learning-based feature selection techniques, clinicians can have access to more comprehensive and objective data to support their diagnostic decision-making process.
3. **Early Detection and Screening:** Deep learning models can be used to analyze imaging data and other clinical features to identify early-stage HCC or even precursor lesions, enabling earlier intervention and potentially improved patient outcomes.
4. **Prognostic Modeling:** Deep learning-based feature selection can be used to develop predictive models for HCC prognosis, helping clinicians stratify patients based on risk and tailor treatment strategies accordingly.
5. **Personalized Treatment Planning**: Deep learning models can assist in identifying specific genetic or molecular markers, as well as imaging features, that can guide the selection of targeted therapies or immunotherapies for individual patients.
6. **Continuous Learning and Improvement:** As more data is collected and used to train and refine deep learning models, the feature selection process can become increasingly accurate and tailored to the specific needs of the healthcare institution or regional population.

### Taxonomy of hepatocellular

Hepatocellular carcinoma (HCC) can be categorized in two ways. One system looks at the cancer cells under a microscope, classifying them as fibrolamellar, pseudoglandular, pleomorphic, or clear cells based on their appearance. A more recent system classifies HCC by its genetic makeup, identifying subtypes like steatohepatitic and scirrhous HCC. This taxonomy helps doctors choose the best treatment and predict patient outcomes. The taxonomy of hepatocellular disease can be approached from two main perspectives: etiology (cause) and pathology.

By Etiology (Cause):

Hepatocellular diseases can arise from a variety of underlying factors, including:

1. Viral Infections:
  Hepatitis B virus (HBV)
  Hepatitis C virus (HCV)
  Hepatitis D virus (HDV)
2. Alcohol Consumption:
  Excessive alcohol intake can result in fatty liver disease, alcoholic hepatitis, and cirrhosis, which may subsequently develop into HCC.
3. Non-alcoholic Fatty Liver Disease (NAFLD):
  NAFLD is characterized by the accumulation of fat in the liver in individuals with little or no alcohol consumption. It can progress to non-alcoholic steatohepatitis (NASH) and cirrhosis, increasing the risk of HCC.
4. Autoimmune Disorders:
  Autoimmune hepatitis, in which the body's immune system attacks the liver, can lead to hepatocellular damage and cirrhosis.
5. Medications and Toxins:
  Certain medications, such as acetaminophen, statins, and antibiotics, as well as exposure to toxins like aflatoxin, can cause direct hepatotoxicity and liver injury.
6. Genetic Factors:
  Inherited genetic predispositions can make individuals more susceptible to developing specific types of hepatocellular diseases.

By Pathology:

The pathological classification of hepatocellular diseases includes:

1. Hepatitis:
  An inflammation of the liver can be caused by viral infections, alcohol, drugs, or autoimmune processes.
2. Cirrhosis:
  A condition characterized by the scarring and hardening of the liver, often resulting from chronic liver diseases like viral hepatitis, NAFLD, or alcoholic liver disease.
3. Hepatocellular Carcinoma (HCC):
  The most common type of primary liver cancer originates from malignant transformation of hepatocytes.
  The World Health Organization (WHO) classification system recognizes several subtypes of HCC, including steatohepatitic, clear cell, macrotrabecular-massive, scirrhous, chromophobe, fibrolamellar, neutrophil-rich, and lymphocyte-rich HCC.

### HCC datasets

Table 1 covers a wide range of HCC datasets, both public and private, that are valuable for research and clinical applications. The public datasets include well-known resources like the LiTS Challenge Dataset, TCGA-LIHC, LiRad Dataset, CPTAC-HCC, DeepLesion, and HCC-LPC. The private datasets, such as BCLC-HCC, HEPAHCC, HCVHCC, and ADEN-HCC, are typically available through collaboration or upon request from the data owners. This table serves as a useful reference for researchers and clinicians interested in accessing HCC datasets for their work, providing a consolidated view of the available resources and their accessibility. It includes the following information:

- Dataset Name: The name of the HCC dataset.
- Description: A brief description of the dataset, including the type and number of samples, and the data modalities (e.g., imaging, genomic, clinical).
- Access: Indicates whether the dataset is public or private. The private datasets are available upon request.
- Link: Provides the URL or source where the public datasets can be accessed.

**Table 1:** A comprehensive table of public and private hepatocellular carcinoma (HCC) datasets

Dataset Name	Description	Access	Link
LiTS (Liver Tumor Segmentation) Challenge Dataset	A dataset of 131 contrast-enhanced CT scans with annotations for liver and liver tumor segmentation	Public	https://competitions.codalab.org/competitions/17094
TCGA-LIHC (The Cancer Genome Atlas—Liver Hepatocellular Carcinoma)	A comprehensive, multi-omics dataset including genomic, transcriptomic, and clinical data for 377 HCC patients	Public	https://portal.gdc.cancer.gov/projects/TCGA-LIHC
LiRad (Liver Imaging Reporting and Data System) Dataset	A dataset of CT and MRI images with annotations based on the LI-RADS classification system for liver lesions	Public	https://www.isi.uu.nl/Research/Databases/LIRADS/
CPTAC-HCC (Clinical Proteomic Tumor Analysis Consortium—Hepatocellular Carcinoma)	A dataset containing proteomic and clinical data for 159 HCC patients	Public	https://proteomics.cancer.gov/data-portal/project/CPTAC-HCC
DeepLesion	A large-scale dataset of 32,735 lesions from 10,594 CT studies, including a subset of HCC lesions	Public	https://nihcc.app.box.com/v/DeepLesion
HCC-LPC (Hepatocellular Carcinoma—Liver Phenotype Classifier)	A dataset of 110 contrast-enhanced CT scans with annotations for HCC and liver phenotypes	Public	https://wiki.cancerimagingarchive.net/display/Public/HCC-LPC
BCLC-HCC (Barcelona Clinic Liver Cancer—Hepatocellular Carcinoma)	A dataset of clinical, radiological, and pathological data for HCC patients treated at the University of Barcelona	Private (available upon request)	http://www.bclc.cat/
Caris Life Sciences	Precision medicine company offering molecular profiling data for HCC	Private (available upon request)	https://www.carislifesciences.com/
Foundation Medicine	Detailed molecular information on cancer, including HCC, derived from clinical specimens	Private (available upon request)	https://www.foundationmedicine.com/
Tempus	Comprehensive cancer data platform providing molecular and clinical data on HCC	Private (available upon request)	https://www.tempus.com/

### Objectives

In this section, we will highlight the key findings and significant contributions that have been made in this work. The review provides a comprehensive analysis of various feature selection techniques used in the context of HCC. It explores traditional methods such as statistical approaches, as well as advanced techniques like deep learning. By comparing and contrasting the strengths and limitations of these methods, the review serves as a valuable resource for researchers and practitioners seeking the most effective approach for feature selection in HCC. One of the major contributions of the review is its focus on deep learning as a promising technique for HCC. It delves into the capabilities of deep learning models, such as convolutional neural networks (CNNs) and recurrent neural networks (RNNs), and their potential to simplify the feature selection process. It highlights the limitations and potential biases associated with current techniques and provides recommendations for future research. By identifying the gaps in existing methodologies, the review offers valuable insights to guide further advancements in feature selection for HCC.

### Reason for scoping review

Deep Learning in Simplifying Feature Selection for Hepatocellular Carcinoma is an important area of research for several reasons:

Improved patient outcomes: Improved accuracy and speed of diagnosis can lead to better patient outcomes, including earlier detection, more effective treatment, and improved survival rates.

Advances in technology: As deep learning algorithms continue to advance [11]; the use of feature reduction techniques is becoming increasingly important. This is because deep learning algorithms are becoming more complex and require more sophisticated feature reduction techniques to achieve optimal performance.

Improved accuracy: Feature reduction techniques can help to improve the accuracy of deep learning algorithms for the detection and diagnosis of hepatocellular carcinoma. By reducing the number of features used in the analysis, the algorithms can be trained more efficiently, leading to better performance.

Faster analysis: Feature reduction techniques can also help to speed up the analysis process, allowing for faster and more efficient detection and diagnosis of hepatocellular carcinoma. This is particularly important in clinical settings where timely diagnosis and treatment can be critical for patient outcomes.

More cost-effective: By reducing the number of features used in the analysis, feature reduction techniques can also make the analysis process more cost-effective. This is important as healthcare systems around the world continue to face financial constraints.

### Organization

We will now briefly describe the organization of the paper. In Sect. 2, we will outline the approach that we used to conduct the review. This will include the methodology we applied, the process for classifying papers, and how we extracted the necessary data. Then, Sect. 3 shows the results of various studies, different methodologies, datasets, and evaluation metrics that have been employed on different methods with deep learning and machine learning approaches for HCC. And the fundamental technical ideas that are necessary for understanding the methods, advantages, and disadvantages of feature selection techniques. Sect. "Methods" introduces a discussion of the Synthesis of findings of the proposed techniques to improve performance and we focus on identifying the main limitations of the review. Finally, some conclusions and open challenges are remarked in Sect. "Discussion".

## Methods

This section focuses on the methodology employed in this scoping review, which encompasses the *Search strategy* and *Study selection*. It outlines the overall process of article selection, as well as the criteria used for inclusion and exclusion.

### Search strategy

This scoping review adopted the PRISMA Extension for Scoping Reviews (PRISMA-ScR) to ensure comprehensive and transparent reporting of the research identification, selection, and analysis. For this review, the literature search was conducted using the Scopus and Web of Science databases. The search utilized keywords such as "Hepatocellular Carcinoma," "Feature selection," "Deep learning," "Machine learning", "feature Reduction," and "Hepatocellular Carcinoma classification or prediction based on clinical variables, histopathology images, and MRI images." To establish eligibility criteria, the search results were filtered based on two factors: the publication timeframe, which required articles to be published between 2013 and 2023, and the article type. This review critically evaluates a corpus of approximately 420 recent scholarly articles obtained from the Scopus and Web of Science databases. These articles are specifically focused on The field of HCC care that focuses on predicting, diagnosing, forecasting, and managing the treatment of HCC patients. By analyzing this substantial body of literature, the review aims to provide a comprehensive understanding of the current state of research in the field and identify key trends, advancements, and challenges in HCC patient management.

### Study selection

**Fig. 3:**
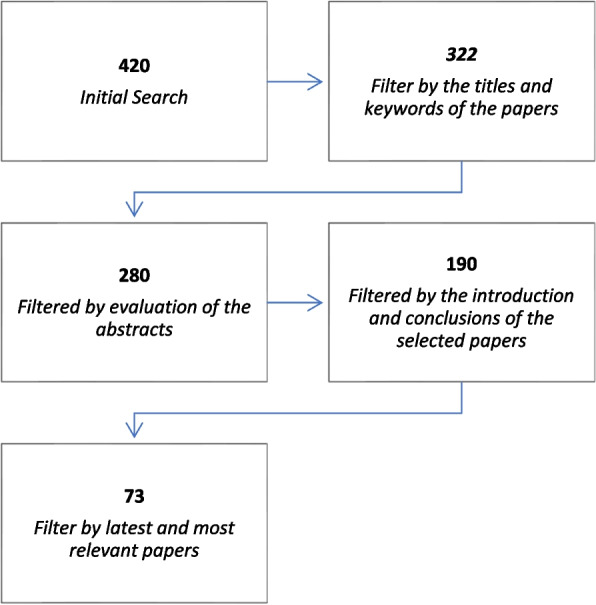
Study selection process

A total of 420 papers were collected from various sources (Fig. 3). These papers underwent a thorough review process from multiple perspectives. Initially, the titles and keywords of the papers were assessed, followed by an evaluation of the abstracts. Finally, the introduction and conclusions of the selected papers were examined. Throughout this process, a snowballing technique was employed to identify additional relevant papers that may have been missed during the initial search. This iterative approach, known as backward snowballing, ensured comprehensive coverage of the literature.

**Fig. 4:**
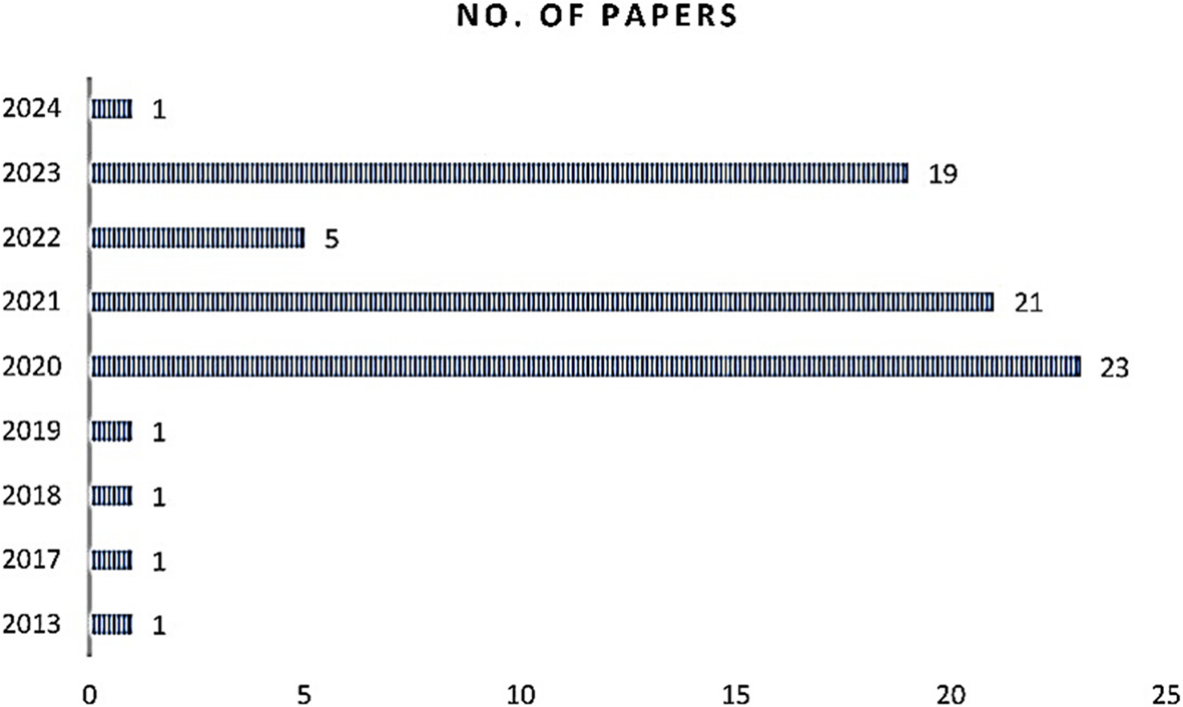
Number of papers selected per year

Upon completion of the review process, a total of 73 papers were identified and selected as potentially valuable contributions (Fig. 4) for the review. These papers were deemed relevant and deemed to contain information that aligns with the objectives of the review.

## Results

In this review, the results were categorized according to the type of HCC Data addressed in the studies. The review categorized available data into three areas: Beginning with clinical data and risk factors, the review then moved on to histopathology slides for diagnosis and prognosis of HCC. Continuing the exploration, the third category delved into imaging techniques like ultrasound, CT, and MRI for further evaluation. We also identified the data source, used algorithms, input, output and key findings that emerged from the analysis. Understanding these findings enhanced the study's impact on the research community.

### HCC clinical data

**Fig. 5:**
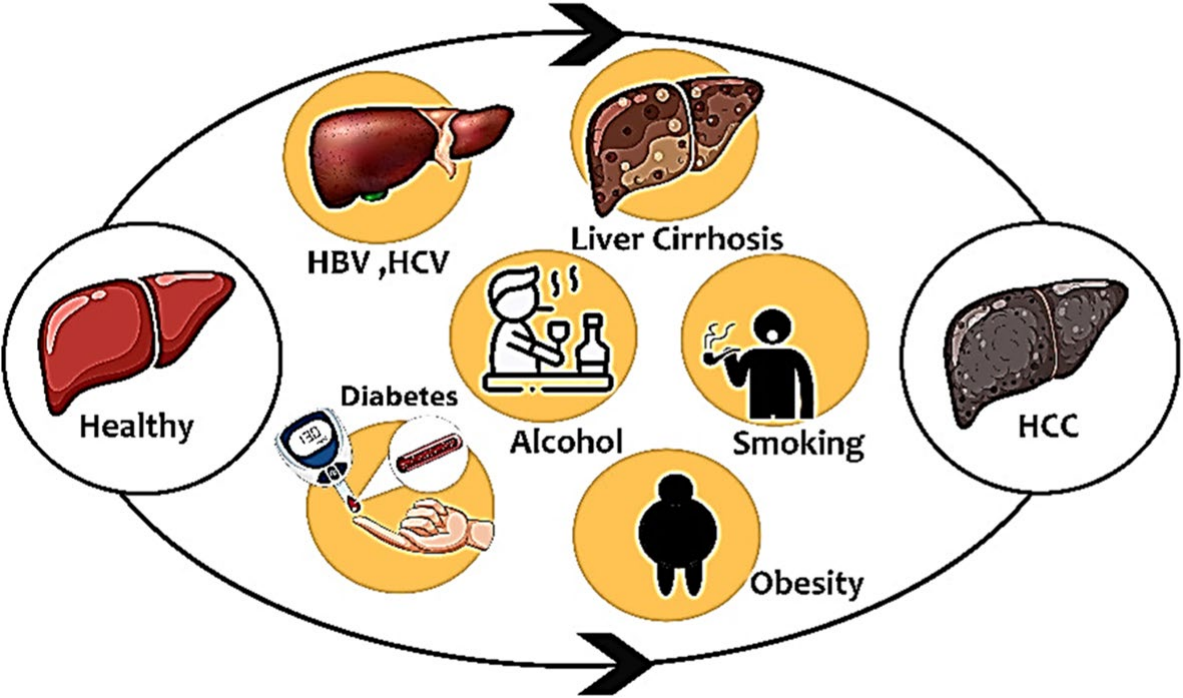
risk factors for HCC development

Clinical data related to HCC includes risk factors for development, symptoms, and signs of disease progression, laboratory values, imaging findings, and a variety of treatment methods. Risk factors for HCC include chronic viral hepatitis, obesity, type 2 diabetes, and exposure to certain chemicals and toxins (Fig. 5). Symptoms of HCC may include abdominal pain, weight loss, jaundice, and fatigue, but many patients may not have symptoms until the disease is advanced. Laboratory values that may be indicative of HCC include elevated levels of alpha-fetoprotein (AFP), alanine aminotransferase (ALT), and aspartate aminotransferase (AST).

Table 2 summarizes several studies that have applied artificial intelligence (AI) techniques to predict the risk of hepatocellular carcinoma (HCC) development using medical data. A wide range of AI models were used, including recurrent neural networks (RNNs), residual networks, convolutional neural networks (CNNs), and support vector machines (SVMs). The studies extracted input data from large clinical databases and electronic health records from centers in Taiwan, Korea, and Portugal. The key output and findings of each study are presented, such as the predictive performance metrics achieved and insights into which patient subgroups were predicted most accurately. This table provides an overview of the progress being made in using advanced analytics and machine learning to personalize HCC risk prediction and help guide clinical decision-making.

**Table 2:** Studies using artificial intelligence (AI) to predict the risk of hepatocellular carcinoma (HCC) based on medical data

Study	Data source	Used algorithms	Input	Output	Key findings
**Ioannou, Tang ** [12]	VHA database	RNN( recurrent neural network)	Clinical Data	Predict HCC development	A recurrent neural network (RNN) model was able to predict the development of HCC with an accuracy of 75.9%, which improved to 80.6% in patients who achieved SVR
**Nam, Sinn ** [13]	Two Korean centers	Residual Network	Clinical Data	Predict HCC development	A Deep learning model for predicting HCC risk achieved better performance than previous models, with an accuracy of 76.3% and AUC of 78.2% in the validation cohort
**Nam, Lee ** [14]	Three Korean centers	ResNet (Residual Network)	Clinical Data	HCC that comes back after a liver transplant	A deep learning (DL) model was significantly better than conventional models at predicting the risk of hepatocellular carcinoma (HCC) recurrence after a liver transplant, with an AUC of 0.75
**Phan, Chan ** [15]	National Health Insurance Research Database of Taiwan	CNN(convolution neural network)	Medical history data	Predict HCC development	A CNN model was able to identify viral hepatitis patients at high risk of HCC development with a high degree of accuracy, as demonstrated by its accuracy of 98.0% and AUC of 0.886
**Ali, Wajahat ** [16]	Coimbra’s Hospital and Universitary Centre (CHUC), Portugal	SVM	Clinical variables	improved hepatocellular carcinoma prediction through dimensionality reduction and genetically optimized support vector machine	The LDA-GA-SVM approach can correctly predict 0.899%
**Deng, Zhu ** [17]	TCGA and HCCDB18 datasets	unsupervised consistent clustering method	clinical features	Comparison of Glycolysis and Cholesterol Gene Expression in Normal and Tumor Samples	The results showed that in the groups of TCGA and HCCDB18 datasets, Normally, cholesterol and glycolytic genes are expressed at lower levels in liver cells than in the four other molecular subtypes of liver cancer. This suggests that the increased expression of these genes is associated with the development of liver cancer
**Cheng, Wang ** [18]	TCGA-LIHC data set	Cox regression analysis	Clinical information and mRNA expression data	Generation and evaluation of the risk model	AUC values of the patient's 3-year and 5-year OS were 0.783 and 0.828, respectively,
**Lv, Li ** [19]	Hospital of Sun Yat-sen University dataset	Random Forest SVM decision tree Logistic regression Neural network Bagging algorithm AdaBoost algorithm	Clinical Data	obtain the prognosis models of three-year tumor-free survival	AUC values of: Logistic regression0.70 Random forest0.71 SVM0.71 C5.0 decision tree 0.70 Neural network 0.67 Bagging algorithm 0.69 AdaBoost algorithm 0.69
**Książek, Gandor ** [20]	Coimbra's Hospital and University Centre	Logistic regression	23quantitative variables and 26qualitative variables	fusion of genetic algorithms for both training the logistic regression model and feature selection	The model using the training set achieved an accuracy equal to: 90.15%
**Liang, Yang ** [21]	National Health Insurance Research Database	CNN	Clinical Data	the convolutional neural network model has immense potential to predict the risk of HCC 1 year in advance with minimal features available in the electronic health records	The area under the receiver operating curve of the model for predicting HCC risk one year in advance was 0.94 (95% CI 0.937–0.943),
**Cao, Fan ** [22]	Shandong Provincial Hospital in China	neighbor2vec logistic regression, KNN, DT, NB, and DNN models	Clinical Data	proposed a new algorithm, neighbor2vec to develop a recurrence prediction model after hepatectomy by combining with a variety of ML algorithms	the accuracy of logistic regression, KNN, DT, NB, and DNN models on the original data set varies from 57.5 to 70.6%, the precision varies from 40.7 to 70.1%, the recall rates change from 20.0 to 67.7%, the FPR ranged from 10.7 to 35.0%, and the standard deviation changes from 0.026 to 0.058. The KNN prediction model outperformed all other models on the original data set, with an accuracy of 70.6%, precision of 70.1%, recall rate of 51.9%, FPR of 16.0%, and standard deviation of 0.042
**Zhang, Lv ** [23]	center in China	3D convolutional neural network	Clinical variables and MR image	analyze MRI images to predict MVI in HCC patients before surgery	The model that ensembles the three MR sequences yielded an AUC of 0.81 (95% CI: 0.74–0.88), sensitivity of 69%, specificity of 79%, and accuracy of 75% in the training set, and an AUC of 0.72 (95% CI: 0.59–0.82), sensitivity of 55%, specificity of 81%, and accuracy of 71% in the validation set
**Mostafa, Mahmoud ** [24]	TCGA	decision trees, Naive Bayes, KNN, neural networks, and SVM	Clinical variables	comparing the performance of different machine learning algorithms in predicting HCC before and after applying feature reduction methods	Following feature reduction, a range of machine learning algorithms including decision trees (96%), Naive Bayes (97.33%), KNN (94.67%), neural networks (96%), and SVM (96.00%) were employed, achieving high predicting accuracies

In a research study conducted by Ioannou, Tang [12], a recurrent neural network (RNN) was trained to predict the development of hepatocellular carcinoma (HCC) within a 3-year timeframe using data from patients with cirrhosis related to hepatitis C virus (HCV). The dataset consisted of 4 variables measured at the beginning of the study and 27 variables measured over time collected from 48,151 patients receiving care through The US Department of Veterans Affairs healthcare system. The results of the study demonstrated that The RNN model was much better than logistic regression at predicting whether patients would develop HCC within the 3-year timeframe. The model was able to predict the development of hepatocellular carcinoma (HCC) with an accuracy of 75.9% in all patients, and 80.6% in patients who achieved sustained virologic response (SVR).

Nam, Sinn [13] conducted a research study in which they developed a deep neural network to forecast the incidence of hepatocellular carcinoma over a 3-year and 5-year period in patients with cirrhosis related to hepatitis B virus (HBV) who were receiving entecavir therapy. The study analyzed 424 patients and demonstrated that the deep learning (DL) model was significantly superior to six other previously reported models that used older approaches to modeling.

techniques. The DL model was also tested on a validation cohort comprising 316 patients, and the results showed that the model achieved a Harrell’s C-index of 0.782, which suggests that it had a high degree of accuracy in predicting the incidence of HCC in these patients**.** In addition to their previous study, Nam, Lee [14] developed a new deep-learning AI-powered model called MoRAL-AI to identify HCC patients at high risk of recurrence after liver transplantation. The model analyzed factors such as the size of the tumor, the patient's age, the level of AFP in the blood, and the prothrombin time to make predictions. Results of the study showed that the MoRAL-AI model was better able to identify HCC patients at high risk of recurrence than traditional models like the Milan, UCSF, up-to-seven, and Kyoto criteria., with a C-index of 0.75 compared to 0.64, 0.62, 0.50, and 0.50, respectively (*P* < 0.001).

In a research study by Phan, Chan [15], a survey was conducted on one million randomly selected Data from Taiwanese health insurance records between 2002 and 2010 to predict the occurrence of liver cancer in patients with viral hepatitis. The medical history of each patient was converted into a 108 × 998 matrix and was then applied to a convolutional neural network to make predictions. The results of the study showed that the CNN model was able to predict liver cancer with an area under the curve of 0.886 and an accuracy of 0.980. The use of CNNsGopinath and Sethuraman [25] for predicting the occurrence of liver cancer in patients with viral hepatitis has significant clinical implications, as early detection of liver cancer can lead to better patient outcomes.

The study of Ali, Wajahat [16] compares the performance of logistic regression, k-nearest neighbors (KNN), decision tree, random forest, and support vector machine (SVM) algorithms in predicting HCC. The study also evaluates the performance of a combination of linear discriminant analysis (LDA), genetic algorithm (GA), and SVM algorithms, which is proposed as a new approach for predicting HCC. The results of the study show that the LDA-GA-SVM approach outperforms the other models, achieving the highest accuracy, sensitivity, and specificity rates. The LDA-GA-SVM approach achieves an accuracy of 0.899, sensitivity of 0.892, and specificity of 0.906, which are higher than the corresponding values obtained using the other models. In another study by Deng, Zhu [17], based on the median standardized expression levels of genes involved in glycolysis and cholesterol production, the samples were divided into four molecular subtypes: Quiescent, Glycolysis, Cholesterol, and Mixed. These subtypes exhibited distinct prognostic differences, with the Mixed subtype having the worst prognosis and the Quiescent subtype showing a favorable prognosis. The Mixed subtype was associated with the activation of cell cycle and oncogenic pathways, while the expression levels of glycolysis and cholesterol production genes were related to the expression levels of genes used for prognostic classification in LIHC.

In a research study by Cheng, Wang [18], To simplify the risk model, a LASSO Cox regression analysis was conducted, resulting in the selection of 23 candidate feature genes. These genes were further subjected to multivariate Cox regression analysis, leading to the identification of 13 optimal feature genes for constructing the risk model. To assess the effectiveness of the model, survival analysis using the Kaplan–Meier method was performed on the training cohort.

The results of the survival analysis demonstrated that patients with low-risk scores had significantly longer survival times compared to those with high-risk scores. To further validate the model, ROC curves were generated, and the corresponding AUC values for the patient's 3-year and 5-year overall survival (OS) were found to be 0.783 and 0.828, respectively. These values indicate that the risk model exhibited effective predictive capabilities for patient outcomes. In this research [19], a total of 50 patients who underwent precision hepatectomy for liver cancer at the Department of Hepatobiliary Surgery of the First Affiliated Hospital of Sun Yat-sen University between June and December 2020 were included. Among these patients, there were 30 males and 20 females, with an average age of 50 years.

The patients included in the study had a precise diagnosis of liver cancer, characterized by an elevated alpha-fetoprotein (AFP) level exceeding 400 μg/L and positive results from one or more dynamic imaging tests. Additionally, they did not have any contraindications for surgery, as assessed by medical professionals [16–18]. None of the patients experienced postoperative recurrence or required planned reoperation. The diameter of the tumors ranged from 1.5 to 20.5 cm, with an average size of 9.6 cm ± 4.9 cm. Before the surgery, the liver function of the patients, as determined by the Child–Pugh grade, was classified as A/B. The retention rate of indocyanine green for 15 min (ICG15 min) was less than 10%, indicating satisfactory liver function. The AFP levels varied between 1 and 58,344 μg/L, with an average value of 7,782.7 μg/L ± 17,573.9 μg/L [20]. In this experiment, a model was developed using the liblinearg gradient algorithm to address the logistic regression weight selection problem. The model had a high success rate in classifying with an accuracy of 78.79%. After 25 iterations, the best result was obtained. By utilizing the training set, the model demonstrated an accuracy rate of 90.15%. A total of 47,945 individuals were included in the study [21], out of which 9,553 were diagnosed with hepatocellular carcinoma (HCC). The model developed for predicting HCC risk one year in advance demonstrated a high level of accuracy, with an area under the receiver operating curve (AUROC) of 0.94 (95% CI 0.937–0.943). The sensitivity of the model was found to be 0.869, indicating its ability to correctly identify individuals at risk of HCC, while the specificity was 0.865, indicating its ability to accurately identify individuals without HCC.

**Fig. 6:**
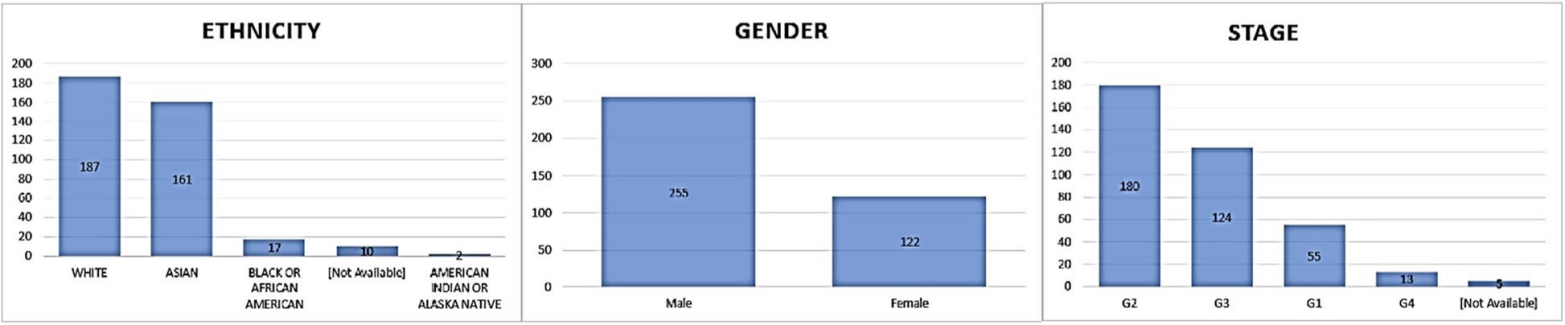
Clinical metadata for Hepatocellular carcinoma samples in TCGA metadata

Furthermore, the AUROC values for predicting HCC patients at different time points in advance were as follows: 0.96 for 7 days, 0.94 for 6 months, 0.94 for 1 year, 0.91 for 2 years, and 0.91 for 3 years. These results demonstrate the model's strong predictive performance at various time intervals, indicating its potential for early detection of HCC cases. Cao, Fan [22] The logistic regression, KNN, DT, NB, and DNN models were evaluated on the original dataset, yielding varying results. The accuracy of these models ranged from 57.5% to 70.6%, while the precision varied between 40.7% and 70.1%. The recall rates ranged from 20.0% to 67.7%, and the false positive rates (FPR) ranged from 10.7% to 35.0%. The standard deviation values ranged from 0.026 to 0.058. Among all the prediction models based on the original dataset, KNN exhibited the best performance. It achieved an accuracy of 70.6%, precision of 70.1%, recall rate of 51.9%, FPR of 16.0%, and a standard deviation of 0.042.Zhang, Lv [23] A total of 237 patients with hepatocellular carcinoma (HCC) were included in the study. Among these patients, 92 (38.8%) were classified as MVI-positive, including 86 males and 6 females, with an average age of 52 ± 12 years. The remaining 145 patients (61.2%) were categorized as MVI-negative, comprising 124 males and 21 females, with an average age of 54 ± 10 years. Patients with MVI exhibited larger tumor size, a higher rate of tumor pseudocapsule, and higher levels of albumin, globulin, and the albumin/globulin ratio compared to patients without MVI (Fig. 6**)**. The best-performing techniques appear to be the deep learning models, particularly the CNN and RNN-based approaches, which achieved high accuracy and AUC values in predicting HCC development and recurrence.

### HCC pathology

DL (Deep Learning) can be effectively applied in the Automatic diagnosis of liver disease from biopsy images, representing a significant field within medical imaging for patients with HCC (Hepatocellular Carcinoma). Not only does DL have the capability to mimic the diagnostic and grading tasks performed by human pathologists with great accuracy [26], but it also offers the potential to identify and analyze intricate imaging features and patterns associated with particular mutations and disease prognosis [27]. This extends the scope of DL models beyond simple replication of human expertise, enabling them to uncover complex information that can enhance our understanding of HCC and its progression.

Table 3 summarizes studies that have applied deep learning and machine learning techniques to analyze liver biopsy images and histopathology slides to diagnose and predict outcomes related to hepatocellular carcinoma (HCC). Convolutional neural networks (CNNs) were commonly employed to extract features from whole-slide images and predict prognosis. Several studies utilized large cancer datasets like The Cancer Genome Atlas (TCGA) to train models. Key outcomes assessed included cancer recurrence after surgery or resection and long-term patient survival. Machine learning algorithms such as support vector machines (SVMs) and artificial neural networks were also applied to clinical and pathological variables. The findings demonstrate how AI-based analysis of tissue samples can outperform conventional scoring systems, with implications for improving HCC management and personalized risk assessment.

**Table 3:** Studies using deep learning and machine learning to analyze liver biopsy images to diagnose, classify, and predict the outcome of hepatocellular carcinoma

Study	Data source	Used algorithms	Input	Output	Key findings
**Yamashita, Long ** [28]	Stanford-HCCDET; TCGA	convolution neural network	Microscopic images of tissue	The reappearance of cancer after surgery	CNN risk scores were better than the TNM system at predicting which patients would have a recurrence of their cancer, and they were also able to identify groups of patients at high and low risk of recurrence
**Saillard, Schmauch ** [29]	French center and TCGA	convolution neural network	Microscopic images	Survival after HCC resection	CNNs using pathology images are a promising new tool for predicting patient survival, outperforming conventional models with a C-index of 0.75–0.78
**Tohme, Yazdani ** [30]	TCGA-LIHC	ANN	Clinic pathological data	used individual patient tumor genomic data to develop a three-gene predictive score	ANN identified 15 genes with normalized importance > 50%
**Saito, Toyoda ** [31]	Yamaguchi University (100 cases), Ogaki Municipal Hospital (47 cases), and Tokyo Medical University (11 cases)	SVM	pathological data	ML-based method for predicting cancer recurrence using all available information on cancer tissue	Prediction used in HCC area information (Accuracy: 88.8%) Prediction used in non-HCC area information (Accuracy: 64.0%)
**Zeng, Zeng ** [32]	Eastern Hepatobiliary Surgery Hospital data	Random survival forests	Clinic pathologic variables	compare the random survival forests (RSF) model with CPH models in the prediction of early recurrence for HCC patients	The time-dependent AUC (2 years) of the RSF model were 0.818 (SE = 0.008), 0.823 (SE = 0.014), and 0.785 (SE = 0.025),
[33]	TCGA	CNN	Microscopic images	classify image patches as containing either HCC or CC	Using a CNN-based 'Liver Cancer Assistant' to accurately differentiate between hepatocellular carcinoma and cholangiocarcinoma. The model had a diagnostic accuracy of 0.885
**Liao, Long ** [34]	TCGA and a center in China	convolution neural network	Microscopic images	HCC detection and prediction of the mutation status of HCC samples	The CNN successfully distinguished hepatocellular carcinoma from adjacent tissues with an AUC of 1.00 and accurately predicted mutations with an AUC exceeding 0.70
**Wang, Jiang ** [35]	TCGA–LIHC	convolution neural network	Microscopic images	Cellular classification of hepatocellular carcinoma	The application of unsupervised clustering revealed the presence of three histological subtypes that complemented molecular pathways and demonstrated prognostic value. The model was accurate in the training dataset, with an overall classification of 99% for tumor cells and 97% for lymphocytes, respectively
**Chen, Zhang ** [36]	GDC online resource center with one center in China	convolution neural network	Microscopic images	Mutations that contribute to HCC progression and metastasis	The CNN achieved an accuracy of 89.6% in predicting tumor differentiation stage and successfully predicted the presence of specific gene mutations
**Lu and Daigle Jr ** [37]	GDC Online data repository	convolution neural network	Microscopic images	Risk of death from HCC	The pre-trained CNN utilized pathology images to predict overall survival (OS) and effectively-identified hepatocellular carcinoma (HCC) subgroups with distinct prognoses
**Shi, Wang ** [38]	1 center in China; TCGA	convolution neural network	Microscopic images	HCC outcomes	The deep learning-based 'tumor risk score' outperformed clinical staging and effectively stratified five groups with varying prognoses

In the study of Yamashita, Long [28], The WSI was preprocessed by removing non-tissue containing a white background using thresholding. Then, they were partitioned into non-overlapping tiles of size 299 × 299 pixels and color normalized. To develop a tumor tile classification model, the Stanford-HCCDET dataset was used, which included WSI with manually annotated tumor regions. This model was applied to each tissue-containing image tile in the TCGA-HCC (*n* = 360 WSI) and Stanford-HCC (n = 198 WSI) datasets to make predictions. The top 100 tiles with the highest predicted probabilities of being tumor tiles were selected and fed into a downstream risk prediction model to generate tile-based risk scores. These scores were then averaged to produce a WSI-level risk score for recurrence. WSI refers to a whole-slide image. Saillard, Schmauch [29] Two deep learning (DL) algorithms were employed using digitized histological slides from 194 patients with hepatocellular carcinoma (HCC) to forecast the survival outcomes of patients who underwent surgical resection. When evaluated on a separate validation set obtained from the Cancer Genome Atlas, both deep learning models demonstrated superior discriminatory capabilities compared to a composite score incorporating all baseline variables linked to patient survival.

The findings of the study Tohme, Yazdani [30] demonstrated the effectiveness of machine learning in predicting survival outcomes in patients with HCC. The model developed using machine learning techniques exhibited strong predictive performance. It successfully stratified patients into different risk groups based on their predicted survival probabilities. Moreover, the model revealed a set of key features and biomarkers that were significantly associated with patient survival, shedding light on the underlying molecular mechanisms of HCC progression. [31]The application of the Support Vector Machine model, trained with a linear kernel, allowed the classification of the Region of Interest in the hepatocellular carcinoma area into three distinct groups, achieving an impressive accuracy of 99.8%. Subsequently, the Region of Interest in the non-HCC area underwent classification using SVM, resulting in a 100% probability score. To assess the reliability of the classification formula derived from the training set, it was validated using the test set. The results indicated that the correct classification probabilities for the ROIs in the HCC were 80.6% and non-HCC areas were 68.1%.

The study of Zeng, Zeng [32]To develop the RFS (Recurrence-Free Survival) model, a total of five hundred survival trees were employed. The Variable Importance (VIMP) analysis revealed that the top five influential factors were tumor size, macrovascular invasion, microvascular invasion, tumor number, and AFP (Alpha-fetoprotein). During the training phase, as well as in the internal and external validation cohorts, the RFS model demonstrated promising performance. The C-index values for the RSF model were 0.725 (SE = 0.005), 0.762 (SE = 0.011), and 0.747 (SE = 0.016), respectively. Additionally, the Gönen & Heller's K statistics for the RSF model were 0.684 (SE = 0.005), 0.711 (SE = 0.008), and 0.697 (SE = 0.014), respectively. Moreover, the time-dependent AUC (2 years) for the RSF model yielded values of 0.818 (SE = 0.008), 0.823 (SE = 0.014), and 0.785 (SE = 0.025), respectively. Kiani, Uyumazturk [33] Employed a CNN-based tool called the "Liver Cancer Assistant," accurate differentiation between hepatocellular carcinoma (HCC) and cholangiocarcinoma was achieved. Remarkably, the model attained a diagnostic accuracy of 0.885, demonstrating its effectiveness in accurately identifying and distinguishing between these two types of liver cancer.

Liao, Long [34], To assess the potential of CNN for predicting the mutation status of HCC samples using solely histopathological images as input, gene mutation data from the TCGA dataset were utilized. The matched Whole Slide Images (WSIs) of HCC samples were used for training the CNN model specifically for task 2 (refer to the Methods section in the Supporting Information for detailed information). During the training phase, only mutations with a minor allele frequency (MAF) of ≥ 10% among the tumors available in the TCGA dataset were selected. This criterion ensured that both the training and test sets comprised a sufficient number of images representing the mutations, enabling robust model training and evaluation. In a study conducted by Wang, Jiang [35], a CNN was trained to Automate the process of identifying and classifying individual nuclei in tissue images using deep learning. This CNN was applied to H&E-stained tissue sections of HCC tumors from the TCGA dataset. Subsequently, feature extraction was performed, resulting in the identification of 246 quantitative image features. Using an unsupervised learning approach, a clustering analysis was carried out. Remarkably, this analysis revealed the presence of three distinct histologic subtypes. Notably, these subtypes were found to be independent of previously established genomic clusters and exhibited varying prognoses. This study showcased the potential of CNN-based image analysis in uncovering unique histologic subtypes that can provide valuable insights into the prognosis of HCC tumors. Chen, Zhang [36] Conducted a study where they trained a CNN to automate the grading of HCC tumors using histopathological H&E images. The CNN exhibited impressive performance, achieving 96% accuracy in differentiating between benign and malignant tumors, and 89.6% accuracy in determining the degree of tumor differentiation. Notably, the CNN was also capable of predicting the identification of certain genetic defects associated with HCC. These results highlight the potential of CNN-based approaches in accurately grading HCC tumors and gaining valuable insights into tumor characteristics and genetic mutations. In their study, Lu and Daigle Jr [37] utilized three pre-trained CNN models to extract imaging features from HCC histopathology samples. They further employed Cox proportional hazards analysis to predict overall survival and disease-free survival. Notably, the study revealed significant correlations between the extracted imaging features and well-established biological pathways. This suggests that the imaging features obtained through the CNN models can provide valuable insights into the prognosis of HCC and its association with underlying biological mechanisms. In their study, Shi, Wang [38] constructed a deep-learning framework utilizing pathologic images from a cohort of 1445 HCC patients. Within this framework, they developed a "tumor risk score" that exhibited prognostic capabilities surpassing clinical staging systems. Remarkably, this DL-based score was found to be independent of existing clinical staging systems and successfully stratified patients into five distinct groups with varying prognoses. The findings highlight the potential of the DL framework in improving prognostic assessments for HCC patients and providing valuable information for personalized treatment strategies.

The best technique used across the studies appears to be convolutional neural networks (CNNs). Several of the studies, such as Yamashita et al. [28], Saillard et al. [29], and Liao et al. [34], utilized convolutional neural networks for tasks like predicting cancer recurrence, survival, and detecting hepatocellular carcinoma (HCC).

### Radiology

Over the past few years, the utilization of AI in New technologies has revolutionized the way we interpret medical images, thanks to the adoption of DL algorithms, particularly Convolutional Neural Network) [30]. These CNN algorithms, trained on diverse imaging modalities such as ultrasound, computed tomography (CT) [39], and magnetic resonance imaging (MRI), have demonstrated exceptional performance in various tasks [40]. They excel in detecting lesions, classifying different types of lesions, accurately segmenting organs and anatomical structures, and even reconstructing high-quality images. The success of DL algorithms in these applications has significantly advanced the field of medical imaging [41], paving the way for enhanced diagnosis and treatment planning [31].

Table 4 provides a brief overview of some common medical imaging modalities used in radiological examinations. Radiology plays a vital role in hepatocellular carcinoma (HCC) diagnosis, staging, treatment planning, and monitoring. The table describes several modalities in terms of their basic operating principles and technologies employed, including ultrasound, computed tomography (CT), magnetic resonance imaging (MRI), positron emission tomography (PET), and single-photon emission computed tomography (SPECT). Each provides unique anatomical or functional information that can aid clinical decision-making. Advanced analytics and artificial intelligence are now helping radiologists extract more insights from these complex datasets. Understanding the range of available modalities is key to appreciating their joint and individual contributions toward improving HCC management.

**Table 4:** Diverse imaging modalities

Imaging Modality	Description
Ultrasound	Uses high-frequency sound waves to produce images of internal structures in real time
Computed Tomography (CT)	Utilizes X-ray technology To produce images of the body's internal structures that can be used to diagnose diseases and other medical conditions
Magnetic Resonance Imaging (MRI)	Utilizes strong magnetic fields and radio waves to create detailed images of the body's internal structures
Positron Emission Tomography (PET)	Involves injecting a radioactive tracer into the body, which emits gamma rays that are detected to produce images
Single-photon emission Computed Tomography (SPECT)	Uses a gamma camera to capture images of the body after injecting a radioactive tracer

Table 5 summarizes studies applying different deep learning and machine learning techniques to radiology images for hepatocellular carcinoma (HCC) diagnosis and prediction. A variety of modalities were utilized, including ultrasound, computed tomography, and magnetic resonance imaging. Common algorithms employed were convolutional neural networks (CNNs) and CNN-based radiomics models. The key outcomes assessed included detection of HCC nodules, classification of focal liver lesions, and prediction of HCC development risk over time. Data sources included single-center cohorts from China, Romania, and the United States. Overall, the deep learning models demonstrated excellent diagnostic performance that approached or exceeded clinicians, highlighting the promise of AI to aid radiologists and improve radiology-based HCC management.

**Table 5:** Radiology-based HCC diagnosis/prediction

Study	Data source	Used algorithms	Input	Output	Key findings
**Jin, Yao ** [42]**2021**	One center in China	Deep learning-based radiomics	Ultrasound images	Predict HCC development	The DL radiomics model successfully predicted the risk of developing hepatocellular carcinoma (HCC) over five years in the test set, achieving a high AUC of 0.900
**Brehar, Mitrea ** [43]	Center in Romania	convolution neural network	Ultrasound images	HCC detection	The CNN model achieved outstanding results in HCC detection, with an AUC of 0.95, accuracy of 91%, 94.4%, and sensitivity of 88.4%
**Shi, Kuang ** [44]	One center in China	convolution neural network	Computed Tomography images	Focal liver lesion type classification	Applying a convolution neural network to three-phase CT images resulted in a noteworthy performance in differentiating hepatocellular carcinoma from other focal liver lesions (FLLs), with an Area Under the Curve (AUC) of 0.925
**Wu, White ** [45]	center in United States	convolution neural network	Magnetic Resonance images	Classification of liver lesions using LI-RADS	The convolution neural network model delivered exceptional results in LI-RADS grading of liver tumors, exhibiting an AUC of 0.95. With an accuracy of 90%, the model demonstrated a high sensitivity of 100% and a positive predictive value (PPV) of 83.5%
**Zhen, Cheng ** [46]	center in China	convolution neural network	Magnetic Resonance images	Liver tumor type classification	The integration of clinical data with a convolution neural network resulted in a highly accurate classification of hepatocellular carcinoma, with an AUC of 0.985 and a strong agreement rate of 91.9% compared to pathology
**Wang, Jian ** [47]	center in China	convolution neural network	Magnetic Resonance images	Microvascular invasion in hepatocellular carcinoma	The combination of deep features from MRI images achieved an AUC of 0.79 when predicting macrovascular invasion (MVI) in HCC patients
**Jiang, Cao ** [48]	center in China	convolution neural network	Computed Tomography images	Microvascular invasion in hepatocellular carcinoma	convolution neural network achieved an AUC of 0.906 for the prediction of MVI. Mean survival was better in the group without MVI
**An, Jiang ** [49]	center in China	convolution neural network	Magnetic Resonance images	detects the amount of normal tissue that is destroyed around a tumor during cancer ablation therapy (ablative margin)	The deep learning model provided accurate estimations of ablative margins and effectively assessed the risk of tumor recurrence at the original site
**Liu, Xu ** [50]	center in China	convolution neural network	Computed Tomography images	Survival rate after transarterial chemoembolization (TACE)	A higher DL score served as an independent prognostic factor, accurately predicting overall survival with AUC values ranging from 0.85 to 0.90
**Liu, Liu ** [51]	center in China	convolution neural network	Ultrasound images	The outcome of Transarterial Chemoembolisation	The model successfully predicted the tumor Outcome of transarterial chemoembolization (TACE) with a remarkable area under the curve of 0.93
**Peng, Kang ** [52]	three centers in China	convolution neural network	Computed Tomography images	The outcome of Transarterial Chemoembolisation	In two separate validation cohorts, the deep learning model exhibited accuracies of 85.1% and 82.8% when predicting the outcome of transarterial chemoembolization (TACE)
**Zhang, Xia ** [53]	three centers in China	convolution neural network	Computed Tomography images	Overall survival of patients treated with transarterial chemoembolization (TACE) and sorafenib	The deep learning signature demonstrated a C-index of 0.714 in accurately predicting overall survival in hepatocellular carcinoma patients who underwent treatment with TACE and sorafenib
**Mitrea, Brehar ** [54]	GE7 dataset and GE9 dataset	fusion between the convolution neural network and CML methods	ultrasound image	enhancing the HCC automatic recognition performance	The integration of CNN-based techniques with traditional machine learning methods, leveraging advanced texture analysis, has demonstrated remarkable effectiveness, yielding classification accuracies surpassing 95% in numerous scenarios
**Lai, Wu ** [55]	China Medical University Hospital	ResNet-18 convolutional neural network	Computed Tomography images and FDG PET-CT images	Overall Survival Prediction in Patients with Hepatocellular Carcinoma Based on 18F-FDG PET-CT Images	The developed prognostic model combined FDG PET-CT images and FDG CT images, Leading to better performance than using CT images alone (0.807 AUC vs. 0.743 AUC). The model utilizing FDG PET-CT images exhibited slightly higher sensitivity than the model relying solely on CT images (0.571 SEN vs. 0.432 SEN)
**Sun, Shi ** [56]	Hospital of Harbin Medical University (Harbin, China)	(LASSO) regression for feature selection Deep learning and radiomic	CECT images	Predicting Treatment Response to Transarterial Chemoembolization (TACE) in Patients with Hepatocellular Carcinoma	The DLRC model was created by incorporating 19 quantitative radiomic features, 10 deep learning features, and 3 clinical factors. In the training cohort, the DLRC model achieved an AUC of 0.937 (95% confidence interval [CI], 0.912–0.962), while in the validation cohort, it achieved an AUC of 0.909 (95% CI, 0.850–0.968)

In the study of Jin, Yao [42], The HCC-R demonstrated superior performance in predicting HCC in the training cohort compared to LSM, GAG-HCC, CAMD, HCC-ESC, and CU-HCC. Comparable findings were observed in the validation and testing cohorts, with HCC-R exhibiting the highest predictive ability for HCC. In the validation cohort, the AUC was 0.942, while in the testing cohort, the AUC was 0.900. The predictive ability of HCC-R was significantly better than that of LSM, GAG-HCC, HCC-ESC, and CU-HCC in the validation cohort, and it was significantly better than that of LSM and CU-HCC in the testing cohort. In a study conducted by Brehar, Mitrea [43], the performance of CNN was compared to conventional machine learning algorithms for hepatocellular carcinoma (HCC) detection using ultrasound images. The CNN model achieved remarkable results, with an AUC of 0.95%, accuracy of 91.0%, sensitivity of 94.4%, and specificity of 88.4%. Notably, The CNN model was superior to the traditional machine learning algorithms in this study.

In the study conducted by Shi, Kuang [44], it was demonstrated that integrating a CNN enabled using three-phase CT imaging to identify HCC protocol with a diagnostic accuracy comparable to that of a four-phase protocol. This advancement offers the potential for patients to receive lower radiation doses during imaging. Moreover, Robust and reliable segmentation of the HCC, liver parenchyma, and other organs in CT images is crucial for determining tumor extent and planning treatment. However, manual contouring of these images is a labor-intensive process and susceptible to variability among different observers. Wu, White [45] constructed a CNN utilizing multiphase MRI images to differentiate between Liver Imaging Reporting and Data System grade 3 and Liver Imaging Reporting and Data System grade 4/5 lesions for hepatocellular carcinoma (HCC) diagnosis. The CNN model achieved an impressive AUC of 0.95 in accurately distinguishing between these grades of lesions. In their study, Zhen, Cheng [46] trained a CNN model that Included unenhanced MRI images and clinical features from 1210 patients with liver tumors. The CNN model exhibited diagnostic performance comparable to that of three experienced radiologists who used enhanced MRI images. Wang, Jian [47] Improved performance was observed in the prediction of MVI (Microvascular Invasion) when employing deep features extracted from b600 images (AUC = 0.74, *p* = 0.004), surpassing the performance of both b0 (AUC = 0.69, *p* = 0.023) and b100 (AUC = 0.734, *p* = 0.011) images. Notably, deep features extracted from the ADC map demonstrated inferior performance (AUC = 0.71, *p* = 0.012) compared to the higher b-value images (b600) in MVI prediction. Combining deep features from b0, b100, b600, and ADC images yielded the most favorable outcomes (AUC = 0.79, *p* = 0.002) for predicting MVI.

Jiang, Cao [48] Out of the 405 patients, 220 (54.3%) were identified as MVI positive, while 185 (45.7%) were classified as MVI negative. The Radiomics-Radiological-Clinical (RRC) Model achieved an area under the receiver operating characteristic curve (AUROC) of 0.952 (95% confidence interval [CI] 0.923–0.973) in the training set, whereas the 3D-CNN Model achieved an AUROC of 0.980 (95% CI 0.959–0.993) in the same set (*p* = 0.14). In the validation set, the AUROC of the RRC Model was 0.887 (95% CI 0.797–0.947), and for the 3D-CNN Model, it was 0.906 (95% CI 0.821–0.960) (*p* = 0.83).

An, Jiang [49] Following a median follow-up period of 28.9 months, 19 patients were identified with local tumor progression (LTP). The mean sizes of the tumor and ablation zone were 2.3 ± 0.9 cm and 3.8 ± 1.2 cm, respectively. Additionally, the mean minimum ablation margin was 3.4 ± 0.7 mm, with a range of 0 to 16 mm. The deformable image registration (DIR) technique exhibited a higher area under the curve (AUC) for 2-year LTP prediction compared to registration assessment without DL, although the difference was not statistically significant (*P* = 0.325).

The rates of LTP at 6, 12, and 24 months were 9.9%, 20.6%, and 24.8% in group A, respectively, while in group B, the rates were 4.0%, 8.4%, and 8.4%, respectively. There were significant differences observed between the two groups (*P* = 0.011). Through multivariate analysis, it was determined that being over 65 years of age (*P* = 0.032, hazard ratio [HR]: 2.463, 95% confidence interval [CI]: 1.028–6.152) and having an ablation margin of ≤ 5 mm (*P* = 0.010, HR: 3.195, 95% CI: 1.324–7.752) were independent risk factors for LTP following microwave ablation (MWA).

In another study Liu, Xu [50] conducted on a cohort of 243 patients with hepatocellular carcinoma who underwent transarterial chemoembolization (TACE), a deep learning (DL) score was developed to predict disease-specific survival based on CT images. The study found that a higher deep learning score was associated with a poor prognosis, with a hazard ratio (HR) of 3.01 and a 95% cumulative incidence (CI) ranging from 2.02 to 4.50. In a study by Liu, Liu [51] 2020, a deep learning (DL) radiomics model was developed to predict the response to trans-arterial chemoembolization (TACE) in 130 patients with hepatocellular carcinoma (HCC) using ultrasound images. The DL model demonstrated high accuracy in predicting TACE response, achieving an AUC of 0.93. The researchers also used the same ultrasound-based DL radiomics model to predict how likely it was that HCC patients would still be alive and the cancer would not have gotten worse after 2 years in a larger group of 419 patients. The goal was to make it easier to choose the best treatment for each patient on the predictions generated by the model. In a study conducted by [52], a convolutional neural network model was trained to predict the outcome of trans-arterial chemoembolization (TACE) using CT images from 562 patients with intermediate-stage hepatocellular carcinoma who were undergoing TACE. The trained model demonstrated high accuracies of 85.1% and 82.8% when evaluated in two validation cohorts. The results suggest the potential of the CNN model to effectively predict TACE response in HCC patients based on CT imaging data. Zhang, Xia [53] developed a deep learning-based model to predict overall survival in 201 people with liver cancer that is not responsive to surgery who received treatment with trans-arterial chemoembolization (TACE) and sorafenib. The DL-based model demonstrated superior predictive performance when compared to the clinical nomogram, with a C-index of 0.730 versus 0.679 (*P* = 0.023). This highlights the potential of the DL model to provide more accurate predictions of overall survival based on CT images in HCC patients undergoing TACE and sorafenib treatment.

The best used technique across the studies is convolutional neural networks (CNNs). The table shows that several studies utilized CNN-based approaches for various tasks related to liver disease diagnosis and characterization, including Predicting the risk of developing hepatocellular carcinoma (HCC) [42]. Detecting HCC [43]. Classifying focal liver lesions [44]. Grading liver lesions using LI-RADS [45]. Classifying liver tumor types [46]. Predicting microvascular invasion in HCC [47, 48].

### Feature selection fundamental

Feature selection is a fundamental step in machine learning and data analysis. It refers to the process of selecting a subset of relevant features or variables from a larger set of available features. The goal of feature selection is to improve model performance, reduce overfitting, enhance interpretability, and reduce computational complexity [57, 58]. Here are some fundamental aspects of feature selection:

- Relevance: Feature selection aims to identify the most relevant features that have a strong relationship with the target variable. Irrelevant or redundant features can introduce noise or unnecessary complexity to the model.
- Dimensionality Reduction: Feature selection helps in reducing the dimensionality of the dataset by selecting a subset of informative features. This is particularly useful when dealing with high-dimensional data, as it can improve model efficiency and reduce computational requirements.
- Overfitting Prevention: Including irrelevant or redundant features can lead to overfitting, where the model becomes too specialized to the training data and performs poorly on unseen data. Feature selection mitigates this risk by focusing on the most informative features, which helps in generalizing the model.
- Interpretability: Feature selection can improve the interpretability of the model by selecting a subset of features that are easily understandable and have a clear relationship with the target variable. This is especially important in domains where interpretability and explainability are crucial, such as healthcare or finance.
- Computational Efficiency: By reducing the number of features, feature selection can significantly improve the computational efficiency of the learning algorithms. This is particularly beneficial when dealing with large-scale datasets or computationally expensive models.
- Data Understanding: Feature selection requires a good understanding of the dataset, including the relationships between features and the target variable. Exploratory data analysis and domain knowledge play important roles in identifying relevant features.
- Evaluation Metrics: The effectiveness of feature selection methods is often evaluated using appropriate metrics such as accuracy, precision, recall, or the area under the receiver operating characteristic curve (AUC-ROC). These metrics help assess the impact of feature selection on model performance.

### Pseudocode

The pseudo-code examples for three commonly used feature selection methods can be presented as follows:



### Feature selection techniques

Various feature selection techniques exist, including filter methods (e.g., statistical measures), wrapper methods (e.g., cross-validation), and embedded methods (e.g., regularization) [59, 60, 61]. Each method has its advantages, limitations, and applicability depending on the dataset and the learning algorithm used. The list of feature selection methods commonly used in machine learning and data analysis can be summarized as follows:

1. Filter Methods: Filter methods evaluate the relevance of each feature independently of the learning algorithm. They use statistical measures or scoring functions to rank features based on their relationship with the target variable. Some commonly used filter methods include:
  Pearson correlation coefficient: Measures the linear correlation between each feature and the target variable. Features with high absolute correlation values are considered more relevant [62].
  Chi-square test: Applicable to categorical target variables, the chi-square test measures the dependence between each feature and the target variable. Features with high chi-square statistics are considered more relevant [63].
  Information gain: Measures the amount of information that a feature provides about the target variable in a decision tree model. Features with high information gain are considered more relevant [64].
  Mutual information: Measures the amount of information shared between a feature and the target variable. Features with high mutual information are considered more relevant [65].
2. Wrapper Methods: Wrapper methods [66] evaluate feature subsets by training and testing a model using different combinations of features. They aim to find the optimal feature subset that maximizes the model's performance. Some commonly used wrapper methods include:
  Recursive Feature Elimination (RFE): Starts with all features and iteratively removes the least important features based on the model's performance. It recursively trains the model on the reduced feature set until a desired number of features is reached. RFE has gained prominence as a feature selection method widely utilized across diverse biomedical disciplines, including [67].
  Forward Selection: Begins with an empty feature set and iteratively adds one feature at a time based on the model's performance until a stopping criterion is met.
  Backward Elimination: Begins with all features and iteratively removes one feature at a time based on the model's performance until a stopping criterion is met.
  Exhaustive Search: Evaluates all possible combinations of features and selects the subset that yields the best model performance. This method can be computationally expensive for large feature sets.
3. Embedded Methods: Embedded methods incorporate feature selection within the model training process. These methods consider the importance of features during model training and select or assign them weights accordingly. Some commonly used embedded methods include:
  L1 regularization (Lasso): Adds a penalty term to the model's cost function, encouraging the model to minimize the coefficients of irrelevant features. Features with non-zero coefficients are considered more relevant. LASSO (L1) regularization facilitates the inclusion of a penalty term that effectively shrinks certain coefficients to zero [68].
  Tree-based feature importance: In tree-based models like Random Forest or Gradient Boosting, features are assigned importance scores based on how much they contribute to the model's accuracy. Features with higher importance scores are considered more relevant.
4. Principal Component Analysis (PCA): PCA is a dimensionality reduction technique that transforms the original features into a lower-dimensional space while preserving the most important information. It identifies orthogonal axes (principal components) that capture the maximum variance in the data and selects a subset of these components as the new features [69].
5. Genetic Algorithms: Genetic algorithms use a population-based approach inspired by the process of natural selection. They generate multiple feature subsets and evaluate their fitness based on the model's performance. The algorithm iteratively evolves the population, applying genetic operations like crossover and mutation, to search for an optimal feature subset [64].
6. Sequential Feature Selection: Sequential feature selection methods evaluate feature subsets by progressively adding or removing features based on a defined evaluation criterion. Some commonly used sequential feature selection methods include:
  Sequential Forward Selection (SFS): Starts with an empty feature set and iteratively adds one feature at a time based on the evaluation criterion until a stopping criterion is met.
  Sequential Backward Selection (SBS): Begins with all features and iteratively removes one feature at a time based on the evaluation criterion until a stopping criterion is met.
7. Correlation-based Feature Selection: This method evaluates the correlation between features and the target variable. It selects features with the highest correlation values, assuming that highly correlated features are more likely to be relevant.
8. Regularization Methods: Regularization techniques, such as L1 (Lasso) and L2 (Ridge) regularization, can be used for feature selection. These methods add a penalty term to the model's cost function, encouraging the model to shrink the coefficients of irrelevant features. Features with non-zero coefficients are considered more relevant.  Information-Theoretic Feature Selection:
9. Information-theoretic feature selection methods measure the amount of information provided by each feature about the target variable. They select features with high information content, such as mutual information, which quantifies the dependence between variables.
10. Relief Methods: Relief methods estimate the importance of features based on their ability to distinguish between instances of different classes. They compute relevance scores for each feature and select features with high scores as relevant [70].

These feature selection methods provide various techniques to identify relevant features, reduce dimensionality, and improve model performance by focusing on the most informative attributes of the data. The choice of method depends on the specific problem, data characteristics, and the learning algorithm being used.

Table 6 summarizes and compares several commonly used feature selection methods along with their key characteristics. Feature selection plays an important role in building efficient machine-learning models by removing irrelevant and redundant features. The table categorizes the methods as filter, wrapper, or embedded based on how they incorporate the learning algorithm. Some popular techniques described include filter methods like statistical tests, wrapper methods using model training/testing, and dimensionality reduction using principal component analysis. The advantages and limitations of each approach are also provided to help guide which technique may be best suited for a given problem based on the dataset properties and modeling goals. Understanding these feature selection fundamentals is important for developing robust AI/ML applications.

**Table 6:** Feature selection methods along with their description, category, advantages, and limitations

Feature Selection Method	Description	Category	Advantages	Limitations
Filter Methods [71, 72]	Evaluate feature relevance independently using statistical measures or scoring functions	Unsupervised	- Computationally efficient - No reliance on the learning algorithm - Can handle high -dimensional data	- Ignores feature dependencies - May not consider interactions with the target variable
Wrapper Methods [73]	Evaluate feature subsets by training and testing the model on different combinations of features	Supervised	- Considers feature interactions - Can optimize model performance - Provides feature importance ranking	- Computationally expensive for large feature sets - Prone to overfitting if the evaluation criterion is not carefully chosen
Embedded Methods [74]	Incorporate feature selection within the model training process by considering feature importance or regularization	Supervised	- Simultaneously performs feature selection and model training - Automatically assigns feature weights - Considers feature interactions	- Limited to specific learning algorithms that support embedded feature selection - May not handle highly correlated features effectively
Principal Component Analysis (PCA) [75]	Dimensionality reduction technique that transforms original features into a lower-dimensional space	Unsupervised	- Reduces dimensionality while preserving important information - Removes multicollinearity among features - Can be used for data visualization	- May lose some interpretability as transformed features are linear combinations of the original features - Assumes linear relationship between features
Genetic Algorithms [76]	Optimization algorithms inspired by natural selection that search for an optimal feature subset	Unsupervised	- Handles large feature spaces - Considers feature interactions - Can handle non-linear relationships	- Computationally expensive - Requires careful parameter tuning - Not guaranteed to find the global optimum

In a previous study [77], a comprehensive review was conducted on various state-of-the-art feature selection methods. The evaluation focused on their effectiveness in addressing common challenges, including correlation and redundancy, data nonlinearity, noise in input features, noise in the target class, and situations where the number of features greatly exceeds the number of samples (as often encountered in microarray datasets). The findings from this study provided valuable insights for practitioners. Table 7 in the study presented practical guidance, based on the specific problem to be addressed, to assist practitioners in selecting appropriate feature selection methods.

**Table 7:** Recommendations for specific feature selection methods for different scenarios [77]. More stars indicate better behavior

Method	Correlation/	Non-	Input	Target	No. Features > >
	Redundancy	linearity	Noise	Noise	No. Samples
CFS	⋆	⋆	⋆	⋆⋆⋆	⋆⋆⋆⋆
Consistency	⋆	⋆	⋆	⋆⋆⋆	⋆⋆
INTERACT	⋆	⋆	⋆	⋆⋆⋆	⋆⋆⋆
InfoGain	⋆	⋆	⋆	⋆⋆⋆	⋆⋆⋆
ReliefF	⋆⋆⋆⋆	⋆⋆⋆⋆⋆	⋆⋆⋆⋆⋆	⋆⋆⋆⋆⋆	⋆⋆
mRMR	⋆⋆⋆⋆	⋆⋆⋆	⋆⋆⋆⋆⋆	⋆⋆	⋆
SVM-RFE^a^	⋆⋆⋆⋆	⋆	⋆	⋆⋆⋆⋆	⋆⋆⋆⋆⋆
SVM-RFE^b^	⋆⋆⋆⋆	⋆⋆⋆⋆⋆	⋆⋆⋆	⋆⋆⋆⋆	–
Wrapper SVM	⋆	⋆	⋆⋆⋆	⋆⋆⋆⋆	⋆⋆
Wrapper C4.5	⋆⋆	⋆⋆⋆	⋆⋆⋆	⋆⋆⋆	⋆⋆⋆

^a^Linear kernel

^b^Non-linear kernel

## Discussion

The results of this review highlight the strong performance of deep learning techniques, particularly convolutional neural networks (CNNs), in addressing a variety of clinical problems related to liver disease. Across the studies evaluated, the CNN-based approaches emerged as the top-performing methods for tasks such as predicting the development and recurrence of hepatocellular carcinoma (HCC), detecting HCC, classifying focal liver lesions, grading liver lesions using LI-RADS, and predicting microvascular invasion in HCC.

Several studies, including the work by Yamashita et al. [28], Saillard et al. [29], and Liao et al. [34], successfully utilized CNN architectures to tackle these clinically relevant challenges. For instance, Jin and Yao [42] developed a CNN model to predict the risk of developing HCC, while Brehar and Mitrea [43] employed CNNs for the detection of HCC. Additionally, Shi and Kuang [44] used CNNs to classify focal liver lesions, and Wu and White [45] leveraged CNNs to grade liver lesions according to the LI-RADS system. Beyond these applications, CNNs have also demonstrated promising results in classifying liver tumor types [46] and predicting microvascular invasion in HCC [47, 48].

This review showed that in the realm of HCC diagnosis, deep learning techniques are unlocking promising avenues for streamlined feature selection. Their ability to harness vast datasets and leverage cutting-edge neural network architectures has led to demonstrably improved accuracy and robustness in identifying predictive features. While machine learning and deep learning have shown promise in various medical applications, including hepatocellular carcinoma (HCC) prediction, there are several limitations associated with their use in this context. Firstly, one major limitation is the requirement for large and high-quality datasets. Machine learning algorithms, including deep learning models, heavily rely on vast amounts of well-curated data to learn patterns and make accurate predictions. However, acquiring such datasets for HCC prediction can be challenging due to the rarity of the disease and the need for comprehensive clinical and imaging data. The limited availability of annotated HCC datasets hampers the development and evaluation of robust models.

Secondly, interpretability and explain ability are crucial in medical decision-making. While deep learning models have demonstrated remarkable predictive capabilities, they often function as black boxes, making it difficult to understand the underlying reasons behind their predictions. This lack of interpretability raises concerns in medical settings, where clinicians need to have confidence in the decision-making process and understand the factors contributing to a prediction.

Thirdly, the generalizability of machine learning and deep learning models can be a limitation. Models trained on specific populations or datasets may not perform as well when applied to different patient populations or settings. The heterogeneity of HCC, including variations in tumor characteristics, genetic profiles, and patient demographics, can introduce challenges in developing models that can effectively predict HCC across diverse populations. Furthermore, the potential for bias in machine learning models is another limitation. Biases can be introduced during the data collection process, such as underrepresentation of certain demographic groups or confounding factors. If the models are trained on biased datasets, they may perpetuate or even amplify existing biases, leading to inaccurate predictions and disparities in healthcare outcomes.

Lastly, the integration of machine learning and deep learning algorithms into clinical practice poses implementation challenges. Deploying these models in real-world healthcare settings requires addressing technical considerations, such as computational resources, integration with existing electronic health record systems, and ensuring robustness and reliability of predictions.

### Study limitations

There are two main limitations of the study:

Expanding the Scope: To further enrich our understanding of this field, future studies could consider including non-English publications and exploring databases beyond Scopus, PubMed, and Web of Science.

Sampling approach: Despite efforts to ensure diverse and meaningful sampling, there's a possibility some relevant studies for review objectives were missed. Future studies employing broader sampling strategies could explore this further.

## Conclusion and future work

This research paper has provided an overview of recent researches on the use of deep learning algorithms for predicting hepatocellular carcinoma (HCC). Through an analysis of various published studies, the paper has reviewed the current state of the art in HCC prediction and highlighted the potential of deep learning algorithms to enhance accuracy and reduce false positives in this field. Moreover, the paper has delved into the challenges associated with using deep learning algorithms for HCC prediction. Challenges such as data availability, model selection, and interpretability have been examined, and potential solutions have been presented.

This study contributes to the growing body of literature on the potential of deep learning algorithms in the field of HCC prediction. However, there is still much to be explored in this area. Therefore, the paper concludes by highlighting potential areas for future research, including the development of more advanced deep learning models, the integration of multi-modal data sources, and the exploration of the ethical implications of using deep learning algorithms in healthcare.

Current models that use clinical variables for predicting hepatocellular carcinoma (HCC) show promise, but there are several areas for future work to further improve their accuracy, personalize risk assessment, and ultimately guide better patient outcomes.

One key area for improvement is the integration of multimodal data. Exploring the combination of clinical data with other modalities, such as genetic information, imaging data (MRI, CT scans), and blood-based biomarkers, could provide a more comprehensive understanding of HCC risk factors. Deep learning models are particularly well-suited for handling such diverse data sources effectively.

Another important direction is to train and validate models on large, geographically diverse datasets. This would help ensure the generalizability of the models and avoid overfitting to specific populations. It is also crucial to account for the presence of other chronic conditions, such as diabetes or viral hepatitis, that may influence the development of HCC.

Additionally, developing models that can incorporate longitudinal data (changes in clinical variables over time) could enable the prediction of risk changes and the earlier identification of high-risk patients. This would allow for more timely interventions and better management of HCC.

By focusing on these future work directions, researchers and clinicians can improve the accuracy and clinical utility of HCC prediction models using clinical variables. This, in turn, could lead to earlier detection, better risk stratification, and ultimately, improved patient outcomes.

## Data Availability

No data was used for the research described in the article.

## Abbreviations

AFP: Alpha-Fetoprotein
AI: Artificial Intelligence
AST: Aspartate Aminotransferase
CFS: Correlation-Based Feature Selection
CHUC: Community Health Urgent Care
CML: Chronic Myeloid Leukemia
DLRC: Deep Learning Radiomics
ESC: Embryonic Stem Cell
FDG: Fluorodeoxyglucose
FLL: Focal Liver Lesion
GAG: Glycosaminoglycan
HBV: Hepatitis B Virus
HCCDET: HCC Detection
HCV: Hepatitis C Virus
LASSO: Least Absolute Shrinkage and Selection Operator
LDA: Linear Discriminant Analysis
LSM: Liver Stiffness Measurement
LTP: Long-Term Potentiation
MAF: Minor Allele Frequency
MVI: Microvascular Invasion
MWA: Microwave Ablation
NAFLD: Non-Alcoholic Fatty Liver Disease
OS: Overall Survival
PPV: Positive Predictive Value
RADS: Radiology
RFE: Recursive Feature Elimination
RFS: Recurrence-Free Survival
RMR: Random Mutation Rule
RRC: Radiomics Radiological Clinical
SBS: Sequential Backward Selection
SFS: Sequential Forward Selection
TACE: Transarterial Chemoembolization
TCGA: The Cancer Genome Atlas
UCSF: University of California, San Francisco
VHA: Veterans’ Health Administration
VIMP: Variable Importance
WSI: Whole Slide Imaging

## References

[1] Bray F, et al. Global cancer statistics 2018: GLOBOCAN estimates of incidence and mortality worldwide for 36 cancers in 185 countries. CA: Cancer J Clin. 2018;68(6):394–424. 30207593 10.3322/caac.21492

[2] Yang JD, et al. A global view of hepatocellular carcinoma: trends, risk, prevention and management. Nat Rev Gastroenterol Hepatol. 2019;16(10):589–604. 31439937 10.1038/s41575-019-0186-y PMC6813818

[3] Stepanova M, et al. Direct and indirect economic burden of chronic liver disease in the United States. Clin Gastroenterol Hepatol. 2017;15(5):759-766. e5. 27464590 10.1016/j.cgh.2016.07.020

[4] Llovet JM, et al. Hepatocellular carcinoma. Nat Rev Dis Primers. 2021;7(1):6. 33479224 10.1038/s41572-020-00240-3 PMC13537433

[5] Yang JD, Heimbach JK. New advances in the diagnosis and management of hepatocellular carcinoma. Bmj. 2020;371:m3544. 33106289 10.1136/bmj.m3544

[6] Ahn JC, et al. Application of artificial intelligence for the diagnosis and treatment of liver diseases. Hepatology. 2021;73(6):2546–63. 33098140 10.1002/hep.31603

[7] Maiti A, Chatterjee B. Improving detection of melanoma and naevus with deep neural networks. Multimedia Tools Appl. 2020;79(21):15635–54.

[8] Maiti A, Chatterjee B, Santosh K. Skin cancer classification through quantized color features and generative adversarial network. Int J Ambient Comput Intell (IJACI). 2021;12(3):75–97.

[9] Bang YH, et al. Clinical relevance of deep learning models in predicting the onset timing of cancer pain exacerbation. Sci Rep. 2023;13(1):11501. 37460584 10.1038/s41598-023-37742-5 PMC10352236

[10] Kaur M, Rattan D. A systematic literature review on the use of machine learning in code clone research. Comput Sci Rev. 2023;47:100528.

[11] Garg A, Mago V. Role of machine learning in medical research: a survey. Comput Sci Rev. 2021;40:100370.

[12] Ioannou GN, et al. Assessment of a deep learning model to predict hepatocellular carcinoma in patients with hepatitis C cirrhosis. JAMA Netw Open. 2020;3(9):e2015626–e2015626. 32870314 10.1001/jamanetworkopen.2020.15626 PMC7489819

[13] Nam JY, et al. Deep learning model for prediction of hepatocellular carcinoma in patients with HBV-related cirrhosis on antiviral therapy. JHEP Reports. 2020;2(6):100175. 33117971 10.1016/j.jhepr.2020.100175 PMC7581930

[14] Nam JY, et al. Novel model to predict HCC recurrence after liver transplantation obtained using deep learning: a multicenter study. Cancers. 2020;12(10):2791. 33003306 10.3390/cancers12102791 PMC7650768

[15] Phan DV, et al. Liver cancer prediction in a viral hepatitis cohort: a deep learning approach. Int J Cancer. 2020;147(10):2871–8. 32761609 10.1002/ijc.33245

[16] Ali L, et al. LDA–GA–SVM: improved hepatocellular carcinoma prediction through dimensionality reduction and genetically optimized support vector machine. Neural Comput Appl. 2021;33:2783–92.

[17] Deng W, et al. Classification and prognostic characteristics of hepatocellular carcinoma based on glycolysis cholesterol synthesis axis. J Oncol. 2022;2022:2014625. 36213830 10.1155/2022/2014625 PMC9546679

[18] Cheng D, et al. Identification and construction of a 13-gene risk model for prognosis prediction in hepatocellular carcinoma patients. J Clin Lab Anal. 2022;36(5):e24377. 35421268 10.1002/jcla.24377 PMC9102505

[19] Lv S, et al. Application of the preoperative assistant system based on machine learning in hepatocellular carcinoma resection. J Healthc Eng. 2021;2021:4757668. 34608411 10.1155/2021/4757668 PMC8487386

[20] Książek W, Gandor M, Pławiak P. Comparison of various approaches to combine logistic regression with genetic algorithms in survival prediction of hepatocellular carcinoma. Comput Biol Med. 2021;134:104431. 34015670 10.1016/j.compbiomed.2021.104431

[21] Liang C-W, et al. Predicting hepatocellular carcinoma with minimal features from electronic health records: development of a deep learning model. JMIR Cancer. 2021;7(4):e19812. 34709180 10.2196/19812 PMC8587326

[22] Cao Y, et al. Prediction model for recurrence of hepatocellular carcinoma after resection by using neighbor2vec based algorithms. Wiley Interdiscip Rev: Data Mining Knowledge Discov. 2021;11(2):e1390.

[23] Zhang Y, et al. Deep learning with 3D convolutional neural network for noninvasive prediction of microvascular invasion in hepatocellular carcinoma. J Magn Reson Imaging. 2021;54(1):134–43. 33559293 10.1002/jmri.27538

[24] Mostafa G, et al. Feature reduction for hepatocellular carcinoma prediction using machine learning algorithms. J Big Data. 2024;11(1):88.

[25] Gopinath M, Sethuraman SC. A comprehensive survey on deep learning based malware detection techniques. Comput Sci Rev. 2023;47:100529.

[26] Baek EB, et al. Application of multiple-finding segmentation utilizing Mask R-CNN-based deep learning in a rat model of drug-induced liver injury. Sci Rep. 2023;13(1):17555. 37845356 10.1038/s41598-023-44897-8 PMC10579263

[27] Shimizu T, et al. A trial deep learning-based model for four-class histologic classification of colonic tumor from narrow band imaging. Sci Rep. 2023;13(1):7510. 37161081 10.1038/s41598-023-34750-3 PMC10169849

[28] Yamashita R, et al. Deep learning predicts postsurgical recurrence of hepatocellular carcinoma from digital histopathologic images. Sci Rep. 2021;11(1):1–14. 33479370 10.1038/s41598-021-81506-y PMC7820423

[29] Saillard C, et al. Predicting survival after hepatocellular carcinoma resection using deep learning on histological slides. Hepatology. 2020;72(6):2000–13. 32108950 10.1002/hep.31207

[30] Tohme S, et al. The use of machine learning to create a risk score to predict survival in patients with hepatocellular carcinoma: a TCGA cohort analysis. Can J Gastroenterol Hepatol. 2021;2021:5212953. 34888264 10.1155/2021/5212953 PMC8651371

[31] Saito A, et al. Prediction of early recurrence of hepatocellular carcinoma after resection using digital pathology images assessed by machine learning. Mod Pathol. 2021;34(2):417–25. 32948835 10.1038/s41379-020-00671-z PMC7817520

[32] Zeng J, et al. Development of a machine learning model to predict early recurrence for hepatocellular carcinoma after curative resection. Hepatobil Surg Nutr. 2022;11(2):176. 10.21037/hbsn-20-466 PMC9023817 35464276

[33] Kiani A, et al. Impact of a deep learning assistant on the histopathologic classification of liver cancer. NPJ Digit Med. 2020;3(1):23. 32140566 10.1038/s41746-020-0232-8 PMC7044422

[34] Liaon H, et al. Deep learning‐based classification and mutation prediction from histopathological images of hepatocellular carcinoma. Clin Transl Med. 2020;10(2):e102. 32536036 10.1002/ctm2.102 PMC7403820

[35] Wang H, et al. Single-cell spatial analysis of tumor and immune microenvironment on whole-slide image reveals hepatocellular carcinoma subtypes. Cancers. 2020;12(12):3562. 33260561 10.3390/cancers12123562 PMC7761227

[36] Chen M, et al. Classification and mutation prediction based on histopathology H&E images in liver cancer using deep learning. NPJ precision oncology. 2020;4(1):14. 32550270 10.1038/s41698-020-0120-3 PMC7280520

[37] Lu L, Daigle BJ Jr. Prognostic analysis of histopathological images using pre-trained convolutional neural networks: application to hepatocellular carcinoma. PeerJ. 2020;8:e8668. 32201640 10.7717/peerj.8668 PMC7073245

[38] Shi J-Y, et al. Exploring prognostic indicators in the pathological images of hepatocellular carcinoma based on deep learning. Gut. 2021;70(5):951–61. 32998878 10.1136/gutjnl-2020-320930

[39] Jiang Y, et al. Biology-guided deep learning predicts prognosis and cancer immunotherapy response. Nat Commun. 2023;14(1):5135. 37612313 10.1038/s41467-023-40890-x PMC10447467

[40] Kushol R, et al. Effects of MRI scanner manufacturers in classification tasks with deep learning models. Sci Rep. 2023;13(1):16791. 37798392 10.1038/s41598-023-43715-5 PMC10556074

[41] Zhang Z, et al. How intra-source imbalanced datasets impact the performance of deep learning for COVID-19 diagnosis using chest X-ray images. Sci Rep. 2023;13(1):19049. 37923762 10.1038/s41598-023-45368-w PMC10624834

[42] Jin J, et al. Deep learning radiomics model accurately predicts hepatocellular carcinoma occurrence in chronic hepatitis B patients: a five-year follow-up. Am J Cancer Res. 2021;11(2):576. 33575088 PMC7868753

[43] Brehar R, et al. Comparison of deep-learning and conventional machine-learning methods for the automatic recognition of the hepatocellular carcinoma areas from ultrasound images. Sensors. 2020;20(11):3085. 32485986 10.3390/s20113085 PMC7309124

[44] Shi W, et al. Deep learning assisted differentiation of hepatocellular carcinoma from focal liver lesions: choice of four-phase and three-phase CT imaging protocol. Abdomin Radiol. 2020;45:2688–97. 10.1007/s00261-020-02485-8 32232524

[45] Wu Y, et al. Deep learning LI-RADS grading system based on contrast enhanced multiphase MRI for differentiation between LR-3 and LR-4/LR-5 liver tumors. Ann Transl Med. 2020;8(11):701. 32617321 10.21037/atm.2019.12.151 PMC7327307

[46] Zhen S-H, et al. Deep learning for accurate diagnosis of liver tumor based on magnetic resonance imaging and clinical data. Front Oncol. 2020;10:680. 32547939 10.3389/fonc.2020.00680 PMC7271965

[47] Wang G, et al. Prediction of microvascular invasion of hepatocellular carcinoma based on preoperative diffusion-weighted MR using deep learning. Acad Radiol. 2021;28:S118–27. 33303346 10.1016/j.acra.2020.11.014

[48] Jiang Y-Q, et al. Preoperative identification of microvascular invasion in hepatocellular carcinoma by XGBoost and deep learning. J Cancer Res Clin Oncol. 2021;147:821–33. 32852634 10.1007/s00432-020-03366-9 PMC7873117

[49] An C, et al. Assessment of ablative margin after microwave ablation for hepatocellular carcinoma using deep learning-based deformable image registration. Front Oncol. 2020;10:573316. 33102233 10.3389/fonc.2020.573316 PMC7546854

[50] Liu Q-P, et al. Prediction of prognostic risk factors in hepatocellular carcinoma with transarterial chemoembolization using multi-modal multi-task deep learning. EClinicalMedicine. 2020;23:100379. 32548574 10.1016/j.eclinm.2020.100379 PMC7284069

[51] Liu F, et al. Deep learning radiomics based on contrast-enhanced ultrasound might optimize curative treatments for very-early or early-stage hepatocellular carcinoma patients. Liver Cancer. 2020;9(4):397–413. 32999867 10.1159/000505694 PMC7506213

[52] Peng J, et al. Residual convolutional neural network for predicting response of transarterial chemoembolization in hepatocellular carcinoma from CT imaging. Eur Radiol. 2020;30:413–24. 31332558 10.1007/s00330-019-06318-1 PMC6890698

[53] Zhang L, et al. Deep learning predicts overall survival of patients with unresectable hepatocellular carcinoma treated by transarterial chemoembolization plus sorafenib. Front Oncol. 2020;10:593292. 33102242 10.3389/fonc.2020.593292 PMC7556271

[54] Mitrea D-A, et al. Hepatocellular carcinoma recognition from ultrasound images using combinations of conventional and deep learning techniques. Sensors. 2023;23(5):2520. 36904722 10.3390/s23052520 PMC10006909

[55] Lai Y-C, et al. Predicting overall survival with deep learning from 18F-FDG PET-CT images in patients with hepatocellular carcinoma before liver transplantation. Diagnostics. 2023;13(5):981. 36900125 10.3390/diagnostics13050981 PMC10000860

[56] Sun Z, et al. Contrast-enhanced CT imaging features combined with clinical factors to predict the efficacy and prognosis for transarterial chemoembolization of hepatocellular carcinoma. Acad Radiol. 2023;30:S81–91. 36803649 10.1016/j.acra.2022.12.031

[57] El Koshiry AM, et al. Detecting cyberbullying using deep learning techniques: a pre-trained glove and focal loss technique. PeerJ Comput Sci. 2024;10:e1961. 38660150 10.7717/peerj-cs.1961 PMC11042001

[58] Maiti A, et al. Computer-aided diagnosis of melanoma: a review of existing knowledge and strategies. Curr Med Imaging. 2020;16(7):835–54. 33059554 10.2174/1573405615666191210104141

[59] Mamdouh Farghaly H, Abd El-Hafeez T. A high-quality feature selection method based on frequent and correlated items for text classification. Soft Comput. 2023;27(16):11259–74.

[60] Mamdouh Farghaly H, Abd El-Hafeez T. A new feature selection method based on frequent and associated item sets for text classification. Concurr Comput: Pract Exp. 2022;34(25):e7258.

[61] Farghaly HM, Ali AA, Abd El-Hafeez T. Building an effective and accurate associative classifier based on support vector machine. Sylwan. 2020;164(3):39–56.

[62] Liu C, et al. LSTM-Pearson gas concentration prediction model feature selection and its applications. Energies. 2023;16(5):2318.

[63] Ahakonye LAC, et al. SCADA intrusion detection scheme exploiting the fusion of modified decision tree and Chi-square feature selection. Internet Things. 2023;21:100676.

[64] Ali W, Saeed F. Hybrid filter and genetic algorithm-based feature selection for improving cancer classification in high-dimensional microarray data. Processes. 2023;11(2):562.

[65] Han F, Wang T, Ling Q. An improved feature selection method based on angle-guided multi-objective PSO and feature-label mutual information. Appl Intell. 2023;53(3):3545–62.

[66] Dakshinamurthy S, Gera BM, Kayarvizhy N. Analytical Performance of Traditional Feature Selection Methods on High Dimensionality Data. In: In 2023 IEEE 8th International Conference for Convergence in Technology (I2CT). 2023.

[67] Liu Y-X, et al. Comparison and development of advanced machine learning tools to predict nonalcoholic fatty liver disease: an extended study. Hepatobil Pancreat Dis Int. 2021;20(5):409–15. 10.1016/j.hbpd.2021.08.004 34420885

[68] Ren S, et al. Preoperative prediction of pathological grading of hepatocellular carcinoma using machine learning-based ultrasomics: a multicenter study. Eur J Radiol. 2021;143:109891. 34481117 10.1016/j.ejrad.2021.109891

[69] Aishwarya R, et al. Parkinson’s Disease Prediction using Fisher Score based Recursive Feature Elimination. In: In 2023 IEEE International Conference on Advancement in Computation & Computer Technologies (InCACCT). 2023.

[70] Masood F, et al. Novel approach to evaluate classification algorithms and feature selection filter algorithms using medical data. J Comput Cogn Eng. 2023;2(1):57–67.

[71] Dai H, et al. Considerable effects of imaging sequences, feature extraction, feature selection, and classifiers on radiomics-based prediction of microvascular invasion in hepatocellular carcinoma using magnetic resonance imaging. Quant Imaging Med Surg. 2021;11(5):1836. 33936969 10.21037/qims-20-218 PMC8047362

[72] Nssibi M, Manita G, Korbaa O. Advances in nature-inspired metaheuristic optimization for feature selection problem: a comprehensive survey. Comput Sci Rev. 2023;49:100559.

[73] Ali MA, et al. A novel method for survival prediction of hepatocellular carcinoma using feature-selection techniques. Appl Sci. 2022;12(13):6427.

[74] Christo VE, et al. Feature selection and instance selection from clinical datasets using co-operative co-evolution and classification using random forest. IETE J Res. 2022;68(4):2508–21.

[75] Tuncer T, Ertam F. Neighborhood component analysis and reliefF based survival recognition methods for Hepatocellular carcinoma. Physica A. 2020;540:123143.

[76] Lee I, et al. Evolutionary learning-derived clinical-radiomic models for predicting early recurrence of hepatocellular carcinoma after resection. Liver Cancer. 2021;10(6):572–82. 34950180 10.1159/000518728 PMC8647074

[77] Bolón-Canedo V, Sánchez-Maroño N, Alonso-Betanzos A. A review of feature selection methods on synthetic data. Knowl Inf Syst. 2013;34:483–519.

